# Muscle Oxygenation During Repeated Double-Poling Sprint Exercise in Normobaric Hypoxia and Normoxia

**DOI:** 10.3389/fphys.2019.00743

**Published:** 2019-06-18

**Authors:** Keiichi Yamaguchi, Nobukazu Kasai, Daichi Sumi, Haruka Yatsutani, Olivier Girard, Kazushige Goto

**Affiliations:** ^1^Graduate School of Sport and Health Science, Ritsumeikan University, Kusatsu, Japan; ^2^Murdoch Applied Sports Science (MASS) Laboratory, Murdoch University, Perth, WA, Australia

**Keywords:** hypoxia, repeated sprints, double-poling, upper limb, muscle oxygenation

## Abstract

We compared upper limb muscle oxygenation responses during repeated double-poling sprint exercise in normobaric hypoxia and normoxia. Eight male kayakers completed a repeated double-poling sprint exercise (3 × 3 × 20-s maximal sprints, 40-s passive recovery, 5-min rest) in either hypoxia (HYP, FiO_2_ = 14.5%) or normoxia (NOR, FiO_2_ = 20.9%). Power output, muscle oxygenation of *triceps brachii* muscle (using near infrared spectroscopy), arterial oxygen saturation, and cardiorespiratory variables were monitored. Mean power output tended to be lower (-5.2%; *P* = 0.06) in HYP compared with NOR, while arterial oxygen saturation (82.9 ± 0.9% vs. 90.5 ± 0.8%) and systemic oxygen uptake (1936 ± 140 vs. 2408 ± 83 mL⋅min^-1^) values were lower (*P* < 0.05). Exercise-induced increases in deoxygenated hemoglobin (241.7 ± 46.9% vs. 175.8 ± 27.2%) and total hemoglobin (138.0 ± 18.1% vs. 112.1 ± 6.7%) were greater in HYP in reference to NOR (*P* < 0.05). Despite moderate hypoxia exacerbating exercise-induced elevation in blood perfusion of active upper limb musculature, power output during repeated double-poling exercise only tended to be lower.

## Introduction

There is a growing interest into the efficacy of repeated sprint training in hypoxia (RSH) on anaerobic capacity and repeated sprint ability (RSA) in team and racket sports (Faiss et al., 2013; Brocherie et al., 2017; Hamlin et al., 2018). RSH consists of repeated “all out” exercise bouts of several seconds (usually ≤30 s) separated with incomplete rest periods (Faiss et al., 2013). It is believed that this novel hypoxic training method may improve RSA primarily through peripheral (e.g., increased blood perfusion and improved fast-twitch fibers behavior) rather than cardiovascular and hematological adaptations (Faiss et al., 2013). According to a recent meta-analysis, RSH causes greater gain for RSA, compared with same training in normoxia, with only trivial differences for maximal oxygen uptake (Brocherie et al., 2017).

Despite previous reports demonstrating improved performance, physiological factors underlying additional training adaptations with RSH have not been fully clarified. Bowtell et al. (2014) compared acute physiological responses (blood lactate concentration and *vastus lateralis* muscle oxygenation) during repeated treadmill sprints under five different levels of inspired oxygen concentrations (FiO_2_). They found that lower FiO_2_ (12–13%) exacerbated exercise-induced elevation in blood lactate and muscle deoxygenation responses. Such elevated blood lactate concentration (Bowtell et al., 2014; Goods et al., 2014) in response to repeated sprint exercise of the lower limbs in hypoxia likely reflects increased anaerobic energy supply via glycolytic pathway. In addition, exercise in hypoxia stimulates nitric oxide production from vascular endothelial cell (Casey et al., 2010), and nitric oxide-induced vasodilatation augments blood perfusion in working muscles (Joyner and Casey, 2014). Such increased blood perfusion enhances shear stress to vascular endothelial cells, leading to vascular endothelial growth factor production, which is known as an angiogenetic growth factor (Dvorak et al., 1995). There are several procedures to evaluate blood perfusion or blood volume in muscles (Gliemann et al., 2018), but the use of doppler ultrasound involves the limitation for monitoring variables during supra-maximal sprint exercise. In contrast, near infrared spectroscopy (NIRS) can be applied during such types of exercise (Smith and Billaut, 2010; Faiss et al., 2015). NIRS data reflect oxygen-related metabolism (oxygenation, deoxygenation) and blood volume in muscles (Hachiya et al., 2008; Truijen et al., 2012). Therefore, assessing muscle oxygenation and muscle blood volume (perfusion) responses using NIRS technology is important to shed more light on physiological factors underpinning RSH efficacy.

To date, the vast majority of previous RSH studies targeted lower limb muscles (e.g., pedaling, running) (Brocherie et al., 2017). While upper limb muscles contain a higher percentage of fast-twitch fibers compared with lower limb muscles (Mygind, 1995; Klein et al., 2003), it was postulated that these fibers are more likely to benefit from RSH than slow-twitch fibers (Faiss et al., 2013). In one of the only available RSH study involving upper limb muscles, Faiss et al. (2015) demonstrated that 2 weeks of repeated double-poling sprint training in hypoxia caused further improvement of RSA. This was accompanied by augmented exercise-induced elevation of blood perfusion (i.e., derived from total hemoglobin level measured non-invasively by NIRS) than the same training in normoxia. In this study, muscle tissue oxygenation has been assessed during a repeated double-poling sprint test to exhaustion (i.e., open-loop test design) conducted in normoxia only. However, the majority of RSH protocols involves the completion of multiple sets of repeated sprint sequences to minimize the likelihood of adopting pacing strategies (Gatterer et al., 2014; Brocherie et al., 2015; Kasai et al., 2015). To date, the time course of acute changes in NIRS-derived variables during a multiple-set, closed-loop RSH exercise of the upper limbs is unknown. Additionally, muscle oxygenation trends during such exercise has never been compared between hypoxia and normoxia in the same individuals.

Therefore, the purpose of the present study was to compare physiological responses to repeated double-poling sprint exercise between normobaric hypoxia and normoxia, with special reference to muscle oxygenation in the upper limbs. We hypothesized that muscle deoxygenation and blood perfusion (i.e., total hemoglobin level) would be augmented during repeated sprint exercise under hypoxic exposure, yet with minimal influence on performance.

## Materials and Methods

### Subjects

Eight male college students were recruited in the present study [mean ± standard error (SE) age, 19.8 ± 0.4 years; height, 174.9 ± 2.4 cm; weight, 71.1 ± 2.2 kg]. All subjects were trained sprint kayakers and were involved in regular training for sprint kayak 6 days/week (2 h/day) on a daily basis with an experience of 4.6 ± 0.7 years. They did not utilize double-poling sprint exercise in daily training, but the subjects had well accustomed to the sprint training using upper limb muscles. Furthermore, a familiarization session using double-poling sprint exercise was conducted before the main experimental trials. All subjects were informed about an experimental overview and possible risks of the present study and gave written informed consent. The present study was approved by the Ethics Committee for Experiments of Ritsumeikan University, Japan.

### Experimental Overview

All subjects visited the laboratory three times throughout the experimental period. On the first visit, a familiarization session for the repeated double-poling sprint exercise was conducted. On the second and third visits, each subject performed the main experimental trial that consisted of completing a repeated double-poling sprint exercise in either acute normobaric hypoxia (HYP, F_i_O_2_ = 14.5%, a simulated altitude of 3000 m) or normoxia (NOR, F_i_O_2_ = 20.9%, sea level) using a randomized, single-blind, cross-over research design. Trials were separated by at least 1 week. All trials (including the familiarization session) were completed in a large (14.8 m^2^) normobaric hypoxic chamber (FCC-5000S; Fuji medical science Co., Ltd., Chiba, Japan) allowing oxygen and carbon dioxide concentrations within the chamber to be continuously monitored. For HYP condition, normobaric hypoxia was created by nitrogen insufflation in the chamber. Power output, muscle oxygenation for *triceps brachii* muscle, and cardiorespiratory variables were evaluated during all sprints. Blood samples were collected to determine changes in blood glucose and lactate concentrations after each of three exercise sets, while exercise-related sensations were evaluated at similar time points.

### Exercise Protocol

Repeated sprint exercise for upper limb muscles was conducted using a double-poling ergometer (SkiErg; Concept 2 Inc., Morrisville, United States). Power was produced by pulling cables spinning a wind resistance flywheel. Subjects were required to pull down the cables using both arms simultaneously with maximal effort and required range motion. In a preliminary session, we have found that it took about 10-s to reach peak power output during maximal sprint due to lower stroke rate and larger range of motion compared with cycle sprint exercise. Therefore, the exercise duration of each sprint was set as 20-s.

**FIGURE 1:**
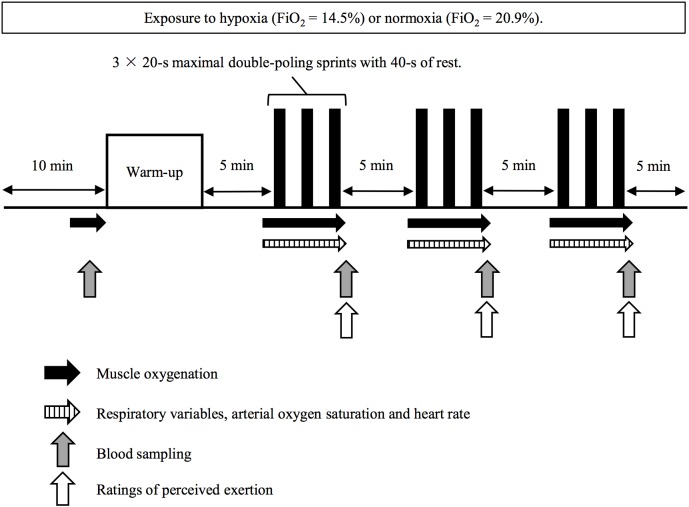
Protocol overview.

On the main experimental trials, subjects rested in a seated position for 10 min (wash-in period) before entry to the chamber, while all equipment was attached. Afterward, they completed a warm-up exercise consisting of 2 min submaximal exercise (1.2 W⋅kg^-1^) followed by two bouts of 10-s maximal sprints. After 5 min of rest, the subjects conducted three sets of 3 × 20-s maximal sprints (nine sprints in total) interspersed with 40 s of passive recovery between sprints with 5 min of rest between sets (Figure 1).

### Measurements

During repeated double-poling sprint exercise, peak and mean power outputs in each 20-s sprint were evaluated. Fatigue index (FI) was calculated using the following equation (Girard et al., 2011) from best (MP_best_) and worst (MP_worst_) mean power output.

$$\mathrm{FI}\;(\%)=100\;\times \;[{\mathrm{MP}}_{\mathrm{best}}(W)-{\mathrm{MP}}_{\mathrm{worst}}(W)]/{\mathrm{MP}}_{\mathrm{best}}(W)$$

Muscle oxygenation variables [oxygenated hemoglobin (oxy-Hb), deoxygenated hemoglobin (deoxy-Hb), and total hemoglobin (total-Hb)] of *triceps brachii* muscle of the right arm were evaluated using a NIRS probe (Hb14; ASTEM Co., Ltd., Kanagawa, Japan) with an inter-optode distance of 30 mm. The NIRS probe was placed on muscle belly of *triceps brachii* muscle (proximal 60% position between acromion and olecranon) and the tissue oxygenation information about 15 mm depth was obtained. These variables were measured before (during 1 min at rest while sitting on a chair before warm-up in each condition) and during each 20-s exercise period (averaged values). All signals were recorded with a sampling frequency of 10 Hz. Because oxy-Hb levels might be confounded by rapid blood volume changes during maximal sprints, only deoxy-Hb and total-Hb were finally analyzed, as previously described (Faiss et al., 2015). In each condition, deoxy-Hb and total-Hb levels were expressed as relative values to resting levels (before exercise).

Cardiorespiratory variables were measured breath-by-breath every 5 s during each 20-s sprint using an automatic gas analyzer (AE-300S; Minato Medical Science Co., Ltd., Tokyo, Japan). Oxygen uptake ($\dot{V}$O_2_), carbon dioxide production ($\dot{V}$CO_2_), minute ventilation ($\dot{V}$E), tidal volume (TV), and respiratory rate (RR) were measured and averaged for each set.

Arterial oxygen saturation (SpO_2_) during each 20-s sprint was monitored continuously every second using a finger pulse oximeter on the right forefinger (PULSOX-Me300; Teijin pharma Ltd., Tokyo, Japan). Heart rate (HR) was also measured continuously (every 5 s) during each 20-s exercise period using a wireless HR monitor (RCX5; Polar Electro, Kempele, Finland). SpO_2_ and HR were presented as average values of all sprints.

Perceived difficulty breathing, upper and lower limbs discomfort were measured using 10-point scales (Christian et al., 2014) immediately after each set of exercise.

Blood samples were collected from a fingertip before warm-up (in each condition) and immediately after each of the three sets of exercise. Blood glucose and lactate concentrations were immediately measured using a glucose analyzer (Free Style; Nipro Corp., Osaka, Japan) and a lactate analyzer (Lactate Pro; Arkray Inc., Kyoto, Japan), respectively.

### Statistical Analysis

All data are presented as the means ± SE. Two-way repeated-measures analysis of variance (ANOVA) was applied using statistical software (SPSS; IBM Corp., Armonk, NY, United States) to assess the main effects of condition, time, and possible interaction between these two factors. When the ANOVA revealed a significant main or interaction effect, the *Tukey-Kramer post hoc* test was performed to identify where differences occurred. Average values of power output, FI, HR, SpO_2_, and RPE were compared between conditions using a paired *t*-test. Effect size was evaluated by partial eta squared (*η*^2^) for two-way ANOVA with repeated measures and Cohen’s *d* for a paired *t*-test. Statistical significance was set at *P* < 0.05.

## Results

### Power Output

**FIGURE 2:**
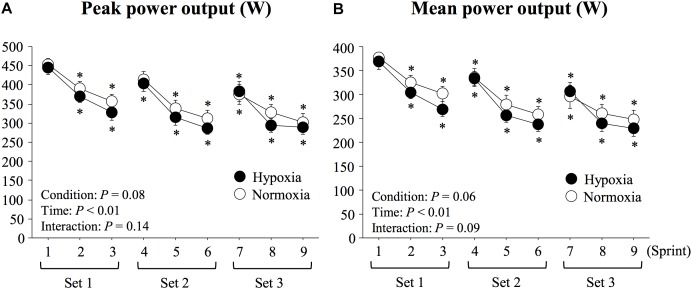
Changes in peak **(A)** and mean power outputs **(B)** during repeated double-poling exercise. Values are means ± SE. ^∗^*P* < 0.05 vs. sprint 1.

Both peak and mean power outputs significantly decreased with time (main effect of time; *P* < 0.01, *η*^2^ = 0.91 for peak power output; *P* < 0.01, *η*^2^ = 0.90 for mean power output, Figure 2). Peak and mean power outputs tended to be lower in HYP compared with NOR, although the differences did not reach statistical significance (main effect of condition; *P* = 0.08, *η*^2^ = 0.37 for peak power output; *P* = 0.06, *η*^2^ = 0.43 for mean power output). In addition, average mean power output (282 ± 15 vs. 298 ± 17 W, *P* = 0.06, *d* = 0.35) of all sprints and FI (38.1 ± 2.9 vs. 35.1 ± 3.5%, *P* = 0.43, *d* = 0.33) did not differ significantly between HYP and NOR. There was no significant condition × time interaction for either peak (*P* = 0.14, *η*^2^ = 0.23) or mean power (*P* = 0.09, *η*^2^ = 0.26) outputs.

### Muscle Oxygenation Variables During Exercise

**FIGURE 3:**
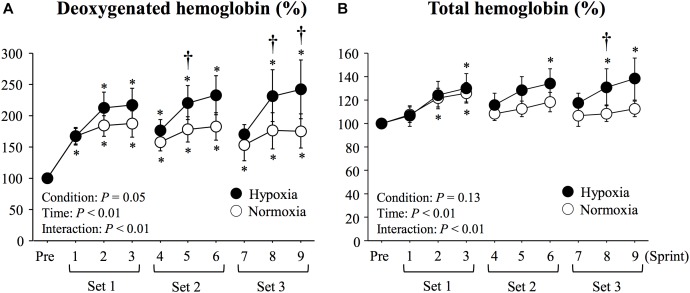
Changes in *triceps brachii* muscle deoxygenated hemoglobin **(A)** and total hemoglobin **(B)** during repeated double-poling exercise. Values are means ± SE. ^∗^*P* < 0.05 vs. Pre. ^†^*P* < 0.05 HYP vs. NOR.

There were significant main effects of condition (*P* = 0.04, *η*^2^ = 0.51), time (*P* < 0.01, *η*^2^ = 0.56), and interaction (*P* < 0.01, *η*^2^ = 0.42) in deoxy-Hb values. with larger elevations seen in HYP than in NOR at sprints 5, 8 and 9 (*P* < 0.05, Figure 3). No significant main effect of condition was observed in total-Hb (*P* = 0.13, *η*^2^ = 0.34). However, there were significant main effect of time (*P* < 0.01, *η*^2^ = 0.43) and interaction (*P* = 0.01, *η*^2^ = 0.32) in total-Hb. Compared with NOR, significantly higher total-Hb values were recorded in HYP at sprint 8 (*P* < 0.05, Figure 3).

### Cardiorespiratory and Perceptual Responses During Exercise

There was a significant main effect of condition (*P* = 0.03, *η*^2^ = 0.54) but not in main effect of time (*P* = 0.11, *η*^2^ = 0.27) and interaction (*P* = 0.67, *η*^2^ = 0.06) in $\dot{V}$O_2_ values. Significant main effects of condition (*P* = 0.04, *η*^2^ = 0.45) and time (*P* < 0.01, *η*^2^ = 0.66) were observed with no significant interaction (*P* = 0.82, *η*^2^ = 0.15) in $\dot{V}$CO_2_ values. $\dot{V}$O_2_ values in sets 1 and 2 together with $\dot{V}$CO_2_ values in set 2 only were significantly lower in HYP vs. NOR (*P* < 0.05, Table 1). There was no significant main effects of condition and time (*P* = 0.10, *η*^2^ = 0.34; *P* = 0.96, *η*^2^ = 0.01, respectively) in $\dot{V}$E. However, significant interaction was observed in $\dot{V}$E (*P* = 0.02, *η*^2^ = 0.37). $\dot{V}$E values in set 1 were significantly higher in HYP vs. NOR (*P* < 0.05, Table 1). No significant main effect of condition (TV, *P* = 0.14, *η*^2^ = 0.29; RR, *P* = 0.89, *η*^2^ < 0.01), time (TV, *P* = 0.40, *η*^2^ = 0.10; RR, *P* = 0.77, *η*^2^ = 0.04), and interaction (TV, *P* = 0.63, *η*^2^ = 0.07; RR, *P* = 0.99, *η*^2^ < 0.01) were observed in both TV and RR.

**Table 1:** Oxygen uptake ($\dot{V}$O_2_), carbon dioxide production ($\dot{V}$CO_2_), minute ventilation ($\dot{V}$E), tidal volume (TV), and respiratory rate (RR) during double-poling sprint exercise.

	Setl	Set 2	Set 3
$\dot{V}$O_2_ (mL⋅min^-1^)			
Hypoxia	1995 ± 149^†^	1908 ± 135^†^	1903 ± 159
Normoxia	2455 ± 109	2444 ± 61	2326 ± 105
$\dot{V}$CO_2_ (mL⋅min^-1^)			
Hypoxia	2758 ± 142	2321 ± 124^†^	2209 ± 112
Normoxia	2865 ± 144	2520 ± 77	2364 ± 113
$\dot{V}$E (L⋅min^-1^)			
Hypoxia	126.6 ± 11.2^†^	121.6 ± 9.4	126.2 ± 10.7
Normoxia	115.9 ± 11.2	120.8 ± 7.2	120.3 ± 9.5
TV (mL⋅breath^-1^)			
Hypoxia	1699 ± 80	1728 ± 62	1635 ± 62
Normoxia	1579 ± 90	1593 ± 50	1556 ± 33
RR (breaths⋅min^-1^)			
Hypoxia	75 ± 6	77 ± 5	77 ± 5
Normoxia	76 ± 7	77 ± 5	78 ± 26


*Values are means ± SE. ^†^*P* < 0.05 HYP vs. NOR.*

Average HR values during exercise did not differ between HYP and NOR (150 ± 3 vs. 146 ± 4 bpm, *P* = 0.32, *d* = 0.28). However, average SpO_2_ values were significantly lower in HYP compared with NOR (82.9 ± 0.9 vs. 90.5 ± 0.8%, *P* < 0.01, *d* = 3.07). Average values of perceived difficulty breathing (9.4 ± 0.2 vs. 9.3 ± 0.3, *P* = 0.28, *d* = 0.17), upper (8.5 ± 0.3 vs. 8.5 ± 0.4, *P* = 0.90, *d* = 0.04), and lower (7.9 ± 0.5 vs. 8.1 ± 0.5, *P* = 0.62, *d* = 0.15) limbs discomfort did not differ between HYP and NOR.

### Blood Variables

There were significant main effects of time in blood glucose (*P* < 0.01, *η*^2^ = 0.76) and lactate concentrations (*P* < 0.01, *η*^2^ = 0.89). Both conditions showed significant increases in blood glucose (after sets 2 and 3) and lactate concentrations (after sets 1, 2, 3), independently of condition (*P* < 0.05) (Table 2). However, no significant main effect of condition (glucose, *P* = 0.33, *η*^2^ = 0.13; lactate, *P* = 0.08, *η*^2^ = 0.37) and interaction (glucose, *P* = 0.65, *η*^2^ = 0.04; lactate, *P* = 0.34, *η*^2^ = 0.15) were observed in blood glucose and lactate concentrations.

**Table 2:** Blood glucose and blood lactate concentrations during double-poling sprint exercise.

	Pre	Set l	Set 2	Set 3
Glucose (mg⋅dL^-1^)			
Hypoxia	90 ± 1	98 ± 3	113 ± 4^∗^	119 ± 6^∗^
Normoxia	91 ± 1	94 ± 2	107 ± 3^∗^	115 ± 8^∗^
Lactate (mmol⋅L^-1^)			
Hypoxia	1.8 ± 0.3	12.6 ± 1.6^∗^	14.2 ± 1.8^∗^	16.9 ± 1.6^∗^
Normoxia	1.8 ± 0.2	11.2 ± 1.1^∗^	12.9 ± 1.3^∗^	14.7 ± 1.3^∗^


*Values are means ± SE. ^∗^*P* < 0.05 vs. Pre.*

## Discussion

The present study compared physiological responses to repeated double-poling sprint exercise of the upper limbs between normobaric hypoxia and normoxia, with special reference to muscle oxygenation. Our main findings indicate that exercise-induced elevations of deoxy-Hb and total-Hb of *triceps brachii* muscle were more profound in HYP compared with NOR, while performance decrements across sprint repetitions did not differ significantly between conditions. In addition, significantly lower $\dot{V}$O_2_ and SpO_2_ occurred in HYP compared with NOR, yet exercise-related sensations as well as blood glucose and lactate concentrations were comparable. Our initial hypothesis stating that acute normobaric hypoxia augments exercise-induced increase of total-Hb value (muscle blood volume) with limited effect on performance during repeated sprint exercise of the upper limbs is therefore verified.

There was no significant difference in repeated double-poling sprint performance between HYP and NOR conditions. Contrastingly, exposure to moderate hypoxia similar to levels used here generally exacerbates fatigability (larger FI) and lowers average performance during repeated sprint exercise of the lower limbs (cycling and running), while initial sprint performance remains unaffected (Girard et al., 2017). These apparent discrepant observations could be due to differences in training background of tested subjects (i.e., competitive athletes), exercise protocol (i.e., longer rest periods between the three sets of sprints), and muscle group recruited (i.e., upper limb muscles) between previous studies using lower limb muscles (Smith and Billaut, 2010; Billaut et al., 2013) and the present one. Nonetheless, statistical analysis revealed that peak and mean power outputs tended to be ∼5% lower (*P* = 0.08 and 0.06, respectively) in HYP vs. NOR. It cannot be ruled out that severer hypoxic conditions, a larger number of sprints completed within each set (only 3 sprints in the present study) and/or a larger number of sets, exaggerating stress on neuromuscular and metabolic regulatory systems, would induce significant differences between conditions (Bowtell et al., 2014; Goods et al., 2014).

The exercise-induced increases in deoxy-Hb and total-Hb across sprint repetitions became progressively larger in HYP vs. NOR. Higher deoxy-Hb levels in HYP are consistent with observations made in a previous study using a 10 × 6-s running sprint exercise (Bowtell et al., 2014). Because total-Hb level evaluated by NIRS reflects blood volume in working muscles (Hachiya et al., 2008; Truijen et al., 2012), augmented hypoxia-induced total-Hb would indicate increased blood perfusion in *triceps brachii* muscle during the exercise in HYP. Our results also show that this effect becomes more visible during the final set of sprints. The greater total-Hb (blood perfusion) in HYP would be explained by compensatory vasodilatation that is intensity dependent (Casey and Joyner, 2012). In an animal study, it is also reported that such vasodilatation would be predominant in fast-twitch fibers (Ferguson et al., 2013). Therefore, the present exercise using maximal sprint and upper limb muscle [containing higher percentage of fast-twitch fibers (Mygind, 1995; Klein et al., 2003)] may accelerate the compensatory vasodilatation. Faiss et al. (2015) reported that 2 weeks of RSH (4 sets of 5 × 10-s maximal sprints) using repeated double-poling exercise caused a further increase in the number of sprints completed until exhaustion compared with the same training in normoxia. In that previous study, total-Hb in *triceps brachii* muscle during the exercise was higher after training for 2-weeks under hypoxic compared with normoxic conditions. It is difficult to directly compare these results with present ones since NIRS data were compared between groups of participants training in different environmental conditions, yet all tested pre- and post-intervention in normoxia. Nonetheless, the authors also suggested that increased number of sprints until exhaustion may be associated with increased blood volume during every exercise session. Collectively, augmented blood perfusion during repeated sprint exercise in HYP is thought to be an important factor for maintaining fatigue resistance (preserved RSA) in the latter phase of exercise.

Oxygen uptake and arterial oxygen saturation values from the first set onward during repeated double-poling sprint exercise were significantly lower in HYP compared with NOR, suggesting an early aerobic energy supply impairment in the O_2_-deprived environment. Ogura et al. (2006) reported that single 40-s sprint exercise in hypoxia (FiO_2_ = 12.7%) caused significant increase in anaerobic energy supply compared with normoxia. In the present study, although $\dot{V}$O_2_ and SpO_2_ were lowered in HYP, peak and mean power outputs did not differ significantly between the two conditions. During “all out” efforts in hypoxia, anaerobic energy supply would be increased to compensate for the decreased aerobic energy supply (Ogura et al., 2006; Morales-Alamo et al., 2012). It was previously reported that maximal running velocity during 20-s maximal running test (Ogawa et al., 2007) and power output during 40-s maximal cycling test (Ogura et al., 2006) did not differ significantly between moderate hypoxia and normoxia. Similarly, in the present study, power output during the maximal double-poling exercise would not be significantly impaired in HYP. In contrast, exercise-induced blood lactate elevations tended to be greater in HYP, suggesting increased anaerobic energy supply.

Although not a universal finding (Willis et al., 2017), a classical observation in the repeated sprint exercise (lower limbs) literature is that exercise-induced blood lactate elevation is augmented in O_2_-deprived environments (Bowtell et al., 2014; Goods et al., 2014). In the present study, blood lactate concentration immediately after exercise tended to be higher in HYP (*P* = 0.08), yet significant difference was not reached between the two conditions. A plausible reason for this apparent lack of significant difference in post-exercise blood lactate concentrations could be related to the training background (sprint kayakers) of tested individuals. Indeed, previous studies that have reported higher post-exercise lactate concentrations in hypoxia vs. normoxia recruited track and field sprinters or team sport athletes (Goods et al., 2014; Morrison et al., 2015).

Practically, the present result suggests that repeated sprint exercise in hypoxia can be applied in athletes having a large solicitation of their upper limb muscles during their sport activities such as skiing, kayaking, judo and wrestling. Larger training adaptations using repeated double-poling sprint exercise in hypoxia vs. normoxia have been already reported (Faiss et al., 2015). However, the present findings add that augmented blood volume during hypoxic exercise would be associated with the greater physiological stimulus to eventually maximize training adaptations. This suggestion may be dependent on the level of hypoxia, because continuous (i.e., exercise and rest periods) exposure to more severe hypoxia levels markedly impairs performance and thereby likely decreases absolute workload during the training session (Bowtell et al., 2014; Goods et al., 2014). Current RSH recommendations indicate that moderate hypoxia (as used in the present study) should be used to maximize training benefits (Brocherie et al., 2017). Finally, several limitations exist in the present study, in particular relatively small sample size. This is because we attempted to recruit homogenous athletes with similar training background who were involved in the similar training program (sprint kayakers at the same university). The comparison of exercise-induced elevation of total-Hb in *triceps brachii* muscle presented large effect size (*η*^2^ = 0.32 for interaction, *η*^2^ = 0.34 for main effect of condition). Although peak and mean power output also presented similar effect size for main effect of condition (peak power output, *η*^2^ = 0.37; mean power output, *η*^2^ = 0.43) and interaction (peak power output, *η*^2^ = 0.23; mean power output, *η*^2^ = 0.26), they did not reach statistical significance. Therefore, further study including large sample size and different training background (e.g., endurance athletes, team sport athletes) may be required to provide robust conclusion for performance difference between the conditions.

## Conclusion

Exercise-induced elevations of deoxy-Hb and total-Hb in *triceps brachii* muscle during repeated double-poling sprint exercise were augmented in hypoxia vs. normoxia, suggesting augmented blood volume in working muscles under these circumstances. However, this was not accompanied by apparent performance difference between conditions across sprint sets, since peak and mean power outputs only tended to be lower in O_2_-derived environment.

## Data Availability

The datasets generated for this study are available on request to the corresponding author.

## Ethics Statement

The present study was approved by the Ethics Committee for Experiments of Ritsumeikan University, Japan.

## Author Contributions

KY and KG conceived and designed the study. KY, NK, DS, and HY conducted the experiments. KY analyzed the data and drafted the manuscript. OG and KG revised the manuscript.

## Conflict of Interest Statement

The authors declare that the research was conducted in the absence of any commercial or financial relationships that could be construed as a potential conflict of interest.
